# Transcriptome analysis revealed SMURF2 as a prognostic biomarker for oral cancer

**DOI:** 10.1007/s13353-024-00869-w

**Published:** 2024-05-03

**Authors:** Lu Deng, Zhihong Wu, Chuanxi Sun, Zhe Liu

**Affiliations:** 1 The Affiliated Stomatological Hospital, Jiangxi Medical College, Nanchang University, Nanchang, Jiangxi Province 330006 China; 2 Jiangxi Province Key Laboratory of Oral Biomedicine, Nanchang, Jiangxi Province 332000 China; 3 Jiangxi Province Clinical Research Center for Oral Diseases, Nanchang, Jiangxi Province 332000 China; 4 Department of Orthodontics, The Affiliated Stomatological Hospital of Jiujiang University, Jiujiang, Jiangxi Province 332000 China

**Keywords:** Immune cell infiltrations, TGF BETA signaling pathway, Oral cavity, Tumor immunity

## Abstract

**Background:**

The activation of TGF-β pathway can facilitate tumorigenesis. Understanding the TGF-related genes (TRGs) in oral cancer and determining their prognostic value is of utmost importance.

**Methods:**

The TRGs were selected to develop a prognostic model based on lasso regression. Oral cancer patients were classified into high-risk and low-risk groups based on the risk model. Subsequently, multivariate COX regression was employed to identify the prognostic marker. Additionally, the expression of SMURF2 was validated using quantitative real-time polymerase chain reaction (qRT-PCR) and the Human Protein Atlas (HPA) database. To investigate the relationship between *SMURF2* expression and immune cell infiltrations, we conducted single-sample Gene Set Enrichment Analysis (ssGSEA) analyses.

**Results:**

We identified 16 differentially expressed TRGs in oral cancer, all of which showed upregulation. From these, we selected eight TRGs as prognostic signatures. Furthermore, the high-risk group demonstrated lower infiltration levels of immune cells, immune score, and higher tumor purity. Interestingly, we also found that *SMURF2* serves as an independent prognostic biomarker. *SMURF2* was upregulated in oral cancer, as confirmed by public databases and qRT-PCR analysis. Importantly, our results indicate a close association between SMURF2 expression and the immune microenvironment.

**Conclusion:**

The 8-TRG signature prognosis model that we constructed has the ability to predict the survival rate and immune activity of oral cancer patients. *SMURF2* could be effective in recognizing prognosis and evaluating immune efficacy for oral cancer.

**Supplementary Information:**

The online version contains supplementary material available at 10.1007/s13353-024-00869-w.

## Introduction

Cancer incidence and mortality rates have been steadily rising globally. In the year 2018 alone, there were an estimated 18.1 million new cases of cancer and 9.6 million cancer-related deaths worldwide. Among these cases, oral cancer made up approximately 2% of the total (Bray et al. 2018; Siegel et al. 2018). The most prevalent form of oral cancer is oral squamous cell carcinoma (OSCC), which is known for its high morbidity and malignancy (Shi et al. 2019; Huang et al. 2019). Oral squamous cell carcinoma (OSCC) constitutes over 90% of all oral tumor cases and is linked to factors including areca nut chewing, immune deficiencies, alcohol and tobacco use, and other related factors (Markopoulos 2012). Despite notable progress in diagnosis and treatment, the survival rate for OSCC remains low at 50% over a 5-year period, while the likelihood of regional recurrence ranges from 33 to 40% (Vigneswaran and Williams 2014; Bello et al. 2010). The unfavorable prognosis of OSCC can be attributed to its highly invasive nature and tendency to metastasize (Jerjes et al. 2010). Furthermore, the TNM classification system, which encompasses the evaluation of the tumor, lymph node, and metastasis, has gained significant popularity and is regarded as an indispensable instrument for prognosticating the survival prospects of individuals with OSCC. Nevertheless, this system has faced criticism due to its inability to decipher the diverse outcomes observed among patients with identical TNM stages (Costa Ade et al. 2005; Mortensen et al. 2022). Therefore, it is of utmost importance to uncover the biological molecular mechanism that drives the progressive of OSCC and to identify new markers that can accurately predict clinical outcomes.

The signaling pathway of transforming growth factor β is involved in numerous vital biological processes, such as cell migration, apoptosis, differentiation and growth, and even the initiation and development of cancer (Shi and Massagué 2003). The TGF-β protein plays a dual role in the formation of tumors. In the early stages of tumor development, it acts as a suppressor, inducing apoptosis and cell cycle arrest to hinder tumor growth. However, as the tumor advances, the tumor cells gradually lose their sensitivity to TGF-β. Consequently, the TGF-β protein released by the tumor cells promotes immune system suppression, facilitates the formation of new blood vessels to nourish the tumor, and enhances the ability of the tumor to invade surrounding tissues and spread to distant sites (Connolly et al. 2012). Many cancers exhibit increased expression of TGF-β and enhanced activation of intracellular signaling through TGF-β receptors (Massagué 2008). It is worth noting that patients with the poorest prognosis show a significant increase in the activity of the TGF-β pathway (Guinney et al. 2015; Calon et al. 2015). Consequently, the activation of this pathway in cancer cells can lead to a process called epithelial-to-mesenchymal transition, in which the normal polarity and adhesion between epithelial cells are lost, and these cells acquire traits of mobile mesenchymal cells (Nieto et al. 2016). The development of oral cancer is caused by the disruption of TGF-β signaling, leading to changes in the normal physiological processes (Prime et al. 2004; Paterson et al. 2001). Upregulation of TGF-β has been observed in samples obtained from patients with oral cancer who have developed metastases to the bones (Takahashi et al. 2020). Furthermore, according to a recent study, it was found that cancer-associated fibroblasts have the ability to secrete TGF-β1, which can enhance OSCC invasion in vitro (Yang et al. 2022). As a result, genes linked to the TGF-β pathway could potentially be used as markers to forecast the prognosis of oral cancer. Nevertheless, there have been insufficient studies examining the connection between TGF-β pathway-related genes (TRGs) and the outcomes of individuals with oral cancer.

**Fig. 1:**
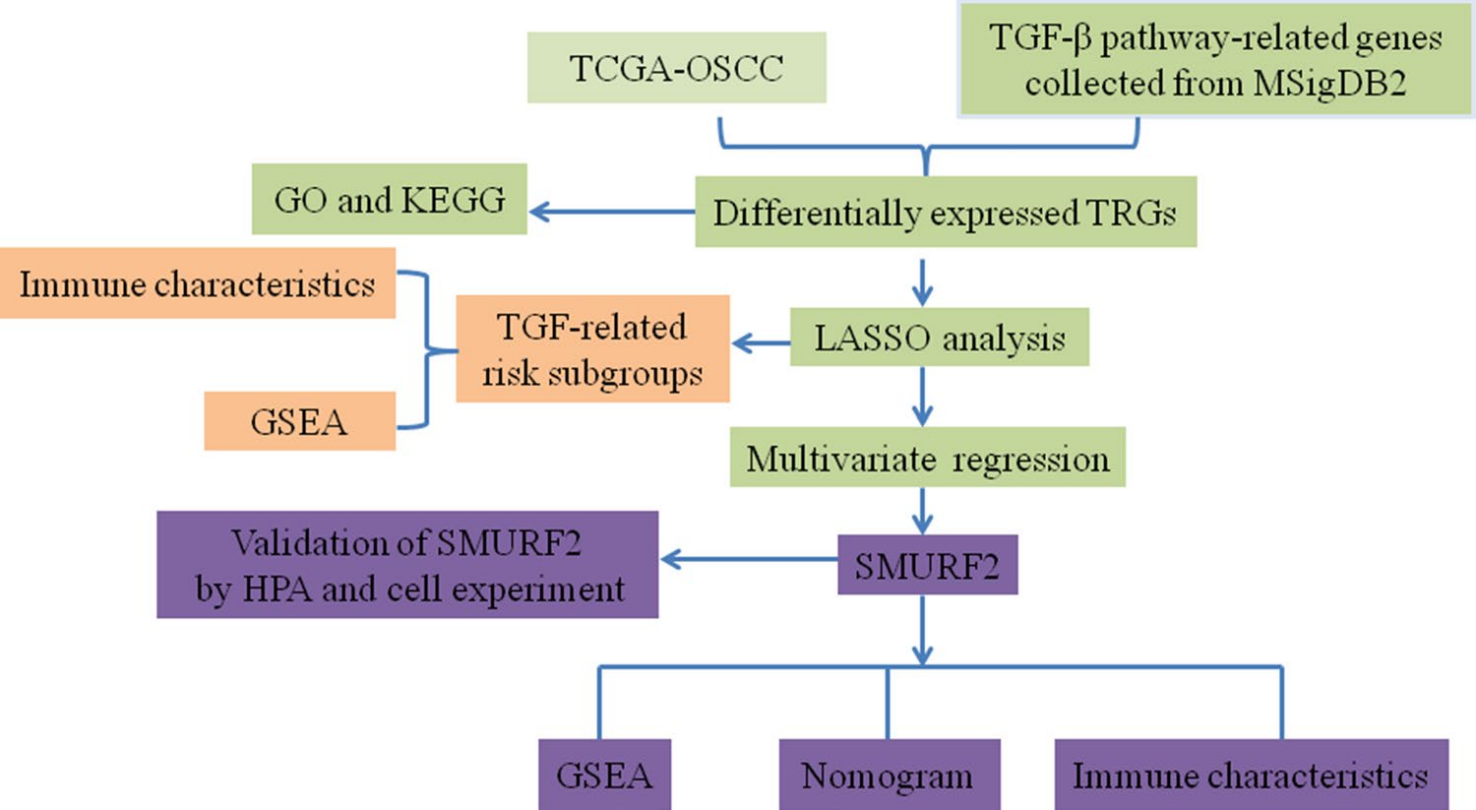
Flowchart outlining the methodology employed in the present study

The emergence of next-generation sequencing technologies and the availability of the Cancer Genome Atlas (TCGA) datasets have made transcriptomic and genomic data on common cancers widely accessible to the public. As a result, this has created an ideal opportunity to analyze and uncover the prognostic and predictive values of potential biomarkers in the field of precision medicine focused on cancer analysis (Fu et al. 2023; Wang et al. 2023; Zhao et al. 2023). The novelty of our work lies in the comprehensive analysis of TRGs in oral cancer, utilizing a robust methodological approach that combines lasso regression for prognostic model development and multivariate Cox regression to identify key prognostic markers. Additionally, by validating the expression of *SMURF2* through quantitative real-time polymerase chain reaction (qRT-PCR) and the Human Protein Atlas database, and examining its relationship with immune cell infiltrations via single-sample Gene Set Enrichment Analysis (ssGSEA), our study not only underscores the prognostic value of *SMURF2* but also illuminates its role in shaping the immune landscape of oral cancer. This study aims to provide a deeper understanding of the TGF-β pathway’s involvement in oral cancer, offering novel insights into the prognostic significance of TRGs and highlighting *SMURF2* as a potential biomarker for prognosis and immune efficacy evaluation in oral cancer. Through our findings, we aspire to contribute to the ongoing efforts to improve the diagnostic precision and therapeutic outcomes for oral cancer patients, paving the way for more personalized and effective cancer care strategies. The flow chart of this study is presented in Fig. 1.

## Methods

### Data collection

The transcriptome expression data of OSCC samples, along with their corresponding clinical data, were downloaded from the TCGA database (https://portal.gdc.cancer.gov) and GEO database (https://www.ncbi.nlm.nih.gov/geo/). We collected a total of 331 OSCC samples and 32 normal samples from the TCGA-OSCC cohort, along with 97 OSCC samples from the GSE41613 dataset. The RNA-seq data in TPM format from TCGA were collected and transformed into log2 [TPM + 1]. TGFP-related genes (TRGs) were collected from the Molecular Signatures Database.

### GSEA

Gene set enrichment analysis (GSEA) is a widely used technique for uncovering the biological mechanisms within clinical specimens by examining the patterns of gene expression they display (Han et al. 2019). We conducted a GSEA using the “ClusterProfiler” package in order to determine the most important pathways within the molecular subgroups. We utilized the “h.all.v7.4.symbols.gmt” subset from the Molecular Signatures Database to assess the relevant pathways and molecular mechanisms. Statistical significance was defined as a *p* value less than 0.05.

### Assessment of the score of TGFP by the GSVA

Gene Set Variation Analysis (GSVA), an unsupervised approach, is utilized to assess the diversity of pathway activity among a group of individuals (Hänzelmann et al. 2013). The GSVA algorithm was applied to calculate the score representing the activity of TGFP. The outcomes are illustrated in the form of box plots.

### Identification of differentially expressed TRGs

To identify differentially expressed TRGs between normal and tumor samples, we utilized the “limma” package. We set the criteria for significance at p.adjust < 0.05 and |log fold change (FC)| ≥ 1 (Fu et al. 2023). The “pheatmap” package was employed to create the heatmap, while the “ggplot2” package was used to generate the volcano plot.

### Enrichment analysis of differentially expressed TRGs

The R package ClusterProfiler was utilized to conduct enrichment analysis for Gene Ontology (GO) and Kyoto Encyclopedia of Genes and Genomes (KEGG). The outcomes were rendered visually through the ggplot2 package. A term was deemed statistically significant if its *p* value was below 0.05.

### Construction of a prognostic signature based on differentially expressed TRGs

We applied LASSO regression analysis to identify survival-related genes. To avoid overfitting, we employed 1000 rounds of cross-validation to select the penalty parameters. By considering the expression levels and coefficient values of prognosis-related genes, we developed a predictive marker for OSCC patients using the following formula: risk score = (expression level of ACVR1*0.1511) − (expression level of BMP2*0.091) + (expression level of CDK9*0.2191) − (expression level of LTBP2*0.0116) + (expression level of SLC20A1*0.1871) + (expression level of SMURF2*0.0042) + (expression level of TGFB1*0.0976) − (expression level of TGFB3*0.1374). The R “Survminer” package was utilized to determine the median risk score, subsequently dividing OSCC patients into high-risk and low-risk groups. The prognostic efficacy of the risk score was assessed by generating a Kaplan-Meier curve using the “survival” package.

### Tumor immune microenvironment analysis

We utilized the ESTIMATE to compare the Stromal Score, Immune Score, and ESTIMATE Score between low-risk and high-risk subgroups (Yoshihara et al. 2013). Additionally, the abundance of immune cells in these subgroups was evaluated through ssGSEA (Huang et al. 2021). The results were visualized using a heatmap and box plot. Furthermore, we examined the correlation between *SMURF2* expression and immune cell infiltration using the ggplot2 package and presented the findings in a lollipop chart.

### Identifying independent prognostic marker

We utilized multivariate Cox regression analysis to further select signature genes. Survival curves were plotted, and the relationship between independent prognostic markers and overall survival was assessed using the ggplot2 and survminer packages. The expression of *SMURF2* in oral cancer was analyzed using the TNMplot.com analysis platform (www.tnmplot.com). In addition, the comparison of SMURF2 protein expression between tumor tissue and normal adjacent tissue was performed using the human protein atlas (HPA) website (https://www.proteinatlas.org).

### Development and assessment of a prognostic nomogram

A predictive tool, known as a nomogram, was created using the results of a multivariate analysis to forecast the overall survival rate of individuals diagnosed with oral cancer. This nomogram was constructed utilizing the ggplot2 and survival R packages, and calibration curves were generated to ensure accuracy. To assess the reliability of the nomogram, ROC curve was employed. Moreover, a decision curve analysis (DCA) was conducted to evaluate the potential clinical utility of the nomogram.

### Cell culture

The oral cancer cell lines (SCC15, SCC9, SCC4, HSC4, and CAL27) and human oral gingival epithelial cell line (HOEC cells) were obtained from the ATCC (Manassas, VA, USA). All cell lines were cultured in high-glucose Dulbecco’s modified Eagle’s medium (Gibco, CA, USA) supplemented with 10% fetal bovine serum and penicillin/streptomycin at 37 °C and 5% CO_2_.

### qRT-PCR

We used Trizol Reagent (Invitrogen, CA, USA) to extract total RNA from cell lines. To assess the purity and concentration of RNA, spectrophotometry at a wavelength of 260/280 nm was employed. Following this, a reverse transcription process was carried out using the reverse transcription kit (Takara). The gene expression levels were detected using SYBR-Green (Takara) and qRT-PCR analysis, with β-ACTIN serving as the internal reference. The primers used are listed in Table S1.

## Results

### Identification of differentially expressed TRGs

**Fig. 2:**
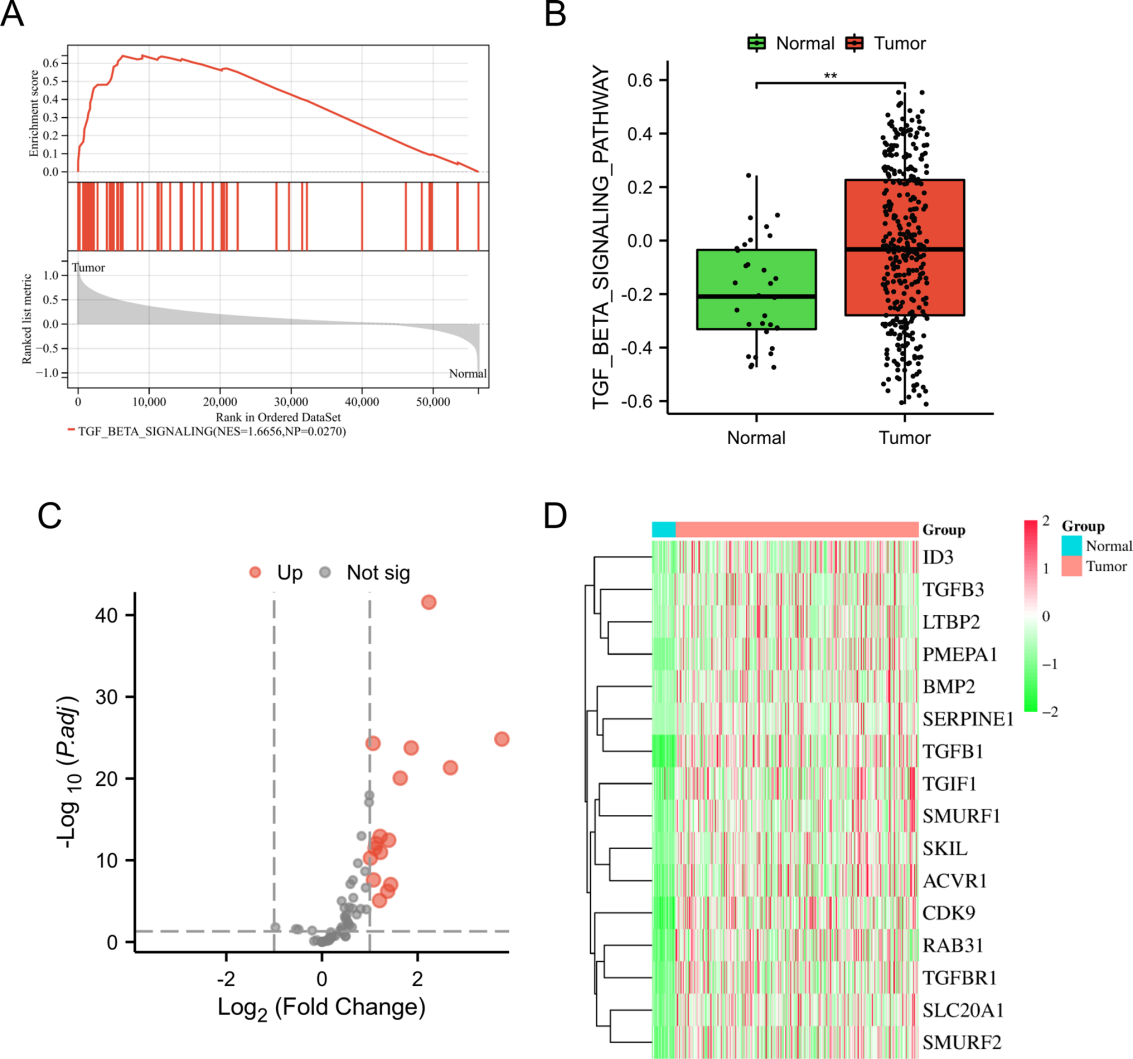
Screening of differentially expressed TRGs. **A** GSEA result showed a significant enrichment of the TGF-β pathway in the tumor group. **B** A box plot was employed to visually illustrate the TGF-β pathway score comparison between the normal group and the oral cancer group. **p < 0.01. **C** The volcano plot depicting the expression level of TRGs. **D** A heatmap was employed to visually display the expression levels of TRGs in both the normal and oral cancer groups

According to the findings in Fig. 2A, the GSEA analysis indicated a significant enrichment of the TGF-β pathway in the tumor group. By employing the GSVA algorithm, we calculated the TGF-β pathway gene set score for each sample. It is important to highlight that the oral cancer group exhibited a significantly higher score for the TGF-β pathway compared to the normal group, as shown in Fig. 2B (*p* < 0.01). These results imply a potential essential role of the TGF-β pathway gene set in the advancement of oral cancer, underscoring the need for further investigation and analysis of this particular gene set. After conducting a comparative analysis between the groups with oral cancer and normal individuals, it was discovered that a total of 16 TRGs exhibited differential expression. These genes were all observed to be upregulated in the tumor group. Specifically, the upregulated genes included *ID3*, *TGFB3*, *LTBP2*, *PMEPA1*, *BMP2*, *SERPINE1*, *TGFB1*, *TGIF1*, *SMURF1*, *SKIL*, *ACVR1*, *CDK9*, *RAB31*, *TGFBR1*, *SLC20A1*, and *SMURF2* (Fig. 2C, D).

### Enrichment analysis of differentially expressed TRGs

**Fig. 3:**
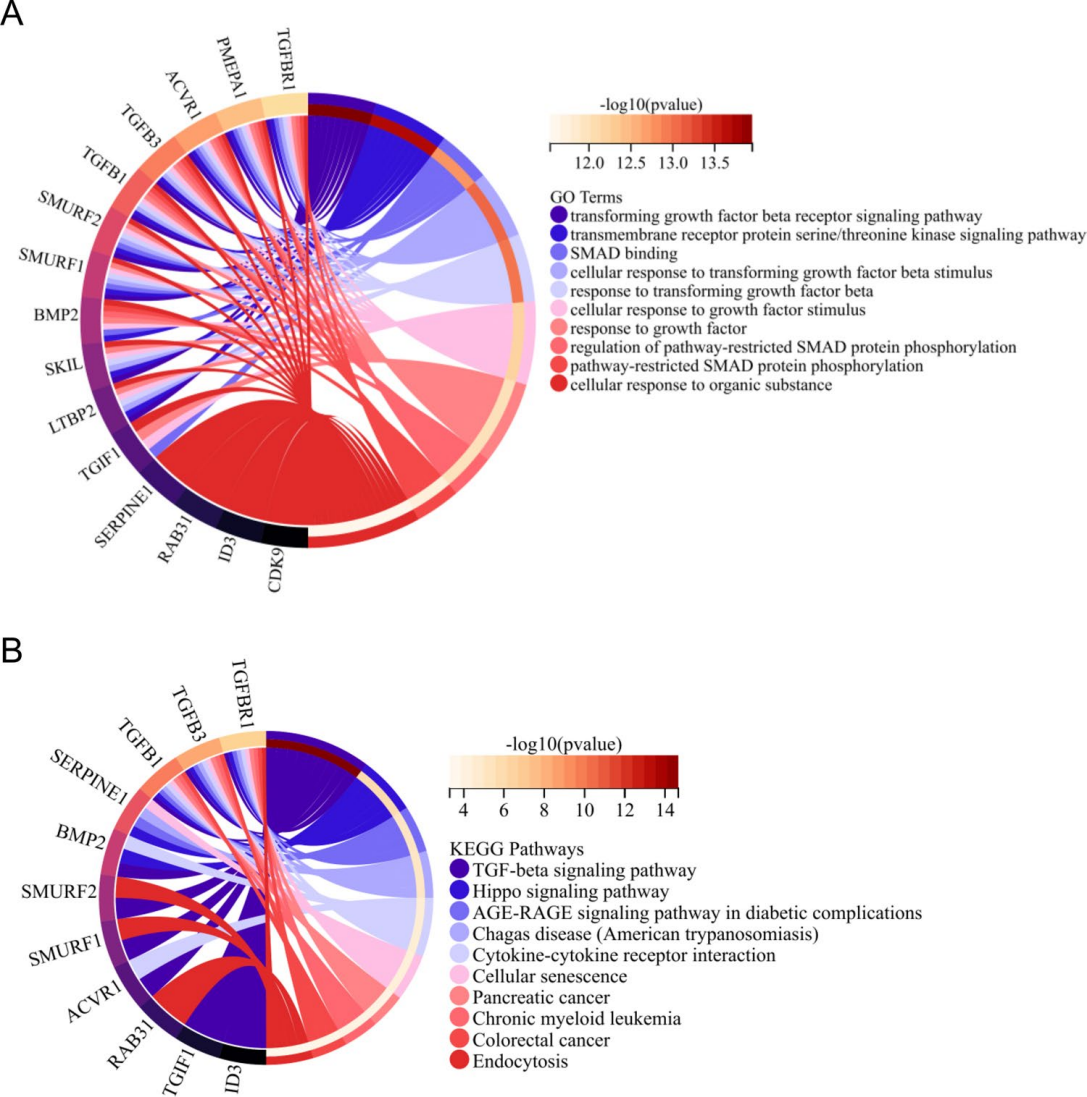
Enrichment analysis of differentially expressed TRGs. Functional annotation analyses were performed using GO enrichment analysis (**A**) and KEGG enrichment analysis (**B**)

As depicted in Fig. 3A, the results from the GO analysis indicated a notable enrichment of these TRGs in the transforming growth factor beta receptor signaling pathway, transmembrane receptor protein serine/threonine kinase signaling pathway, SMAD binding, cellular response to transforming growth factor beta stimulus, etc. KEGG analysis revealed that these TRGs were significantly enriched in the TGF-beta signaling pathway, hippo signaling pathway, cytokine-cytokine receptor interaction, cellular senescence, etc. (Fig. 3B).

### Establishment of a prognostic signature related to TRGs

**Fig. 4:**
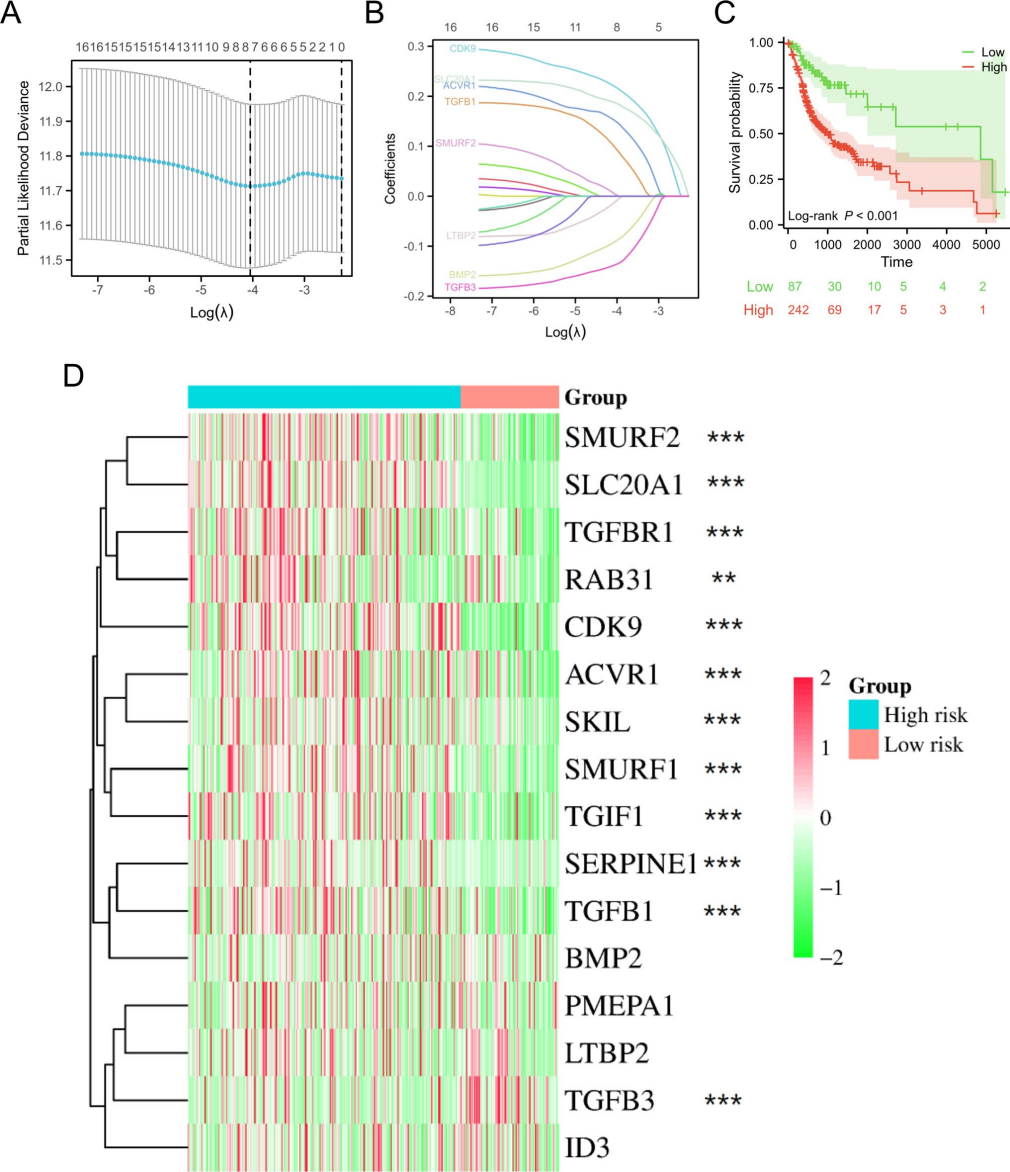
Establishment of a prognostic signature related to TRGs. **A**, **B** LASSO variable trajectory plots were analyzed to identify non-zero variables. **C** Comparison of survival outcomes between OSCC patients classified as high risk and low risk in the TCGA-OSCC dataset. **D** A heatmap was employed to visually display the expression levels of TRGs in both the low-risk and high-risk subgroups. **p < 0.01; ***p < 0.001

The LASSO regression analysis was performed using the previously mentioned 16 TRGs. Screening of the data revealed eight TRGs (*TGFB3*, *LTBP2*, *BMP2*, *TGFB1*, *ACVR1*, *CDK9*, *SLC20A1*, and *SMURF2*) with non-zero coefficients, indicating their association with the prognosis of oral cancer patients (Fig. 4A, B). The Kaplan-Meier curve demonstrated that patients with OSCC who were classified as high risk experienced significantly reduced overall survival compared to those classified as low risk (Fig. 4C). Furthermore, it was observed that a significant proportion of TRGs were upregulated in the subgroup with a high risk (Fig. 4D).

### Immuno-infiltration analysis of the TRG-related signature

**Fig. 5:**
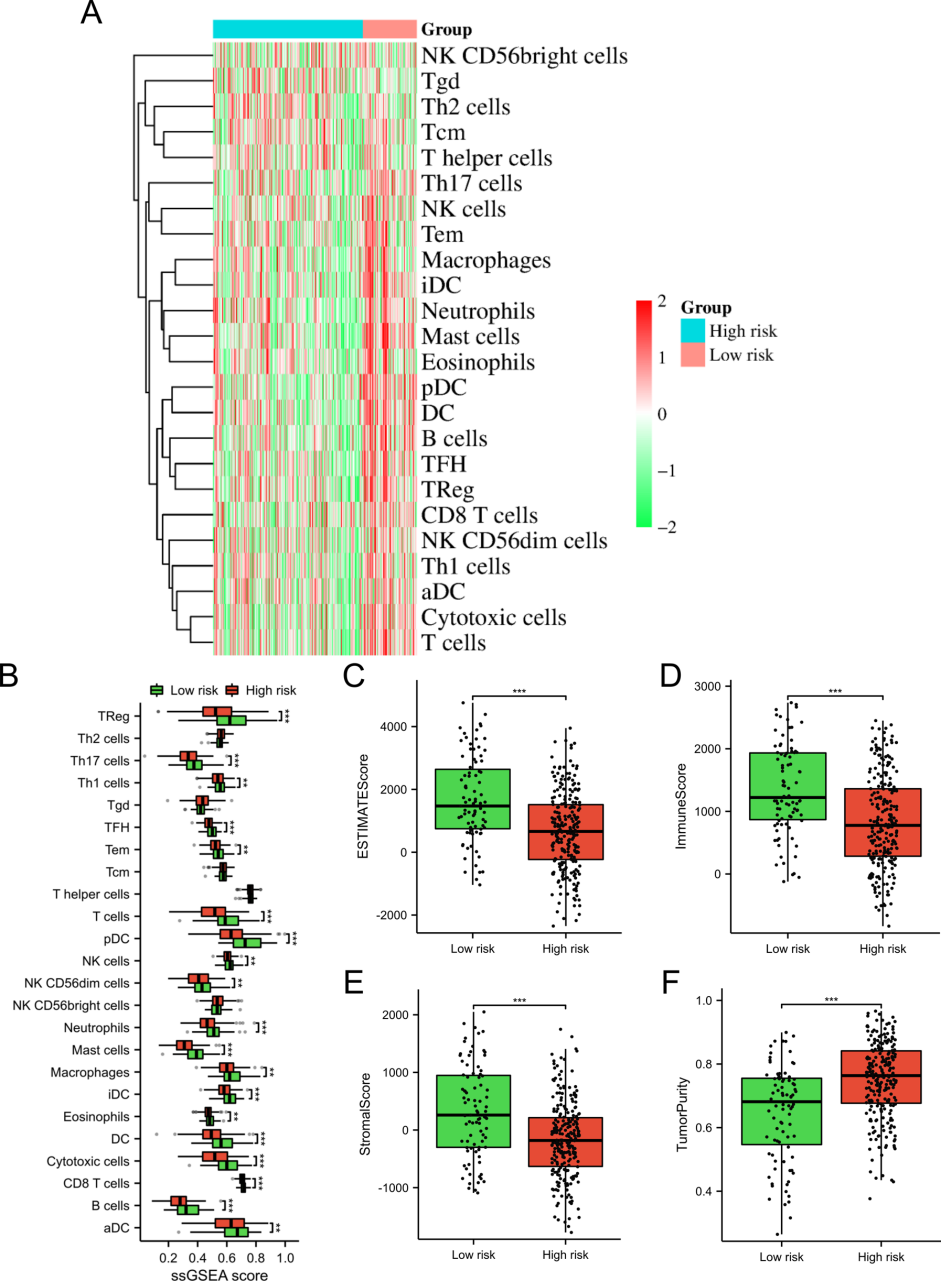
Immune infiltration analysis. We utilized the heatmap (**A**) and box plot (**B**) to visually illustrate the enrichment fraction of infiltrating cells in both high-risk and low-risk subgroups. Variations in the ESTIMATEScore (**C**), ImmuneScore (**D**), StromalScore (**E**), and tumor purity (**F**) among patients with high-risk and low-risk statuses. **p < 0.01; ***p < 0.001

By utilizing ssGSEA, we investigated the association between the risk score and immune infiltration and found that patients with a lower risk score showed increased infiltration of immune cells in comparison to those with a higher risk score (Fig. 5A, B). By conducting ESTIMATE algorithm, we demonstrated that the low-risk group had higher ESTIMATE score, stromal score, and immune score, whereas the low-risk group had lower tumor purity compared to the high-risk group (Fig. 5C–F).

### GSEA

**Fig. 6:**
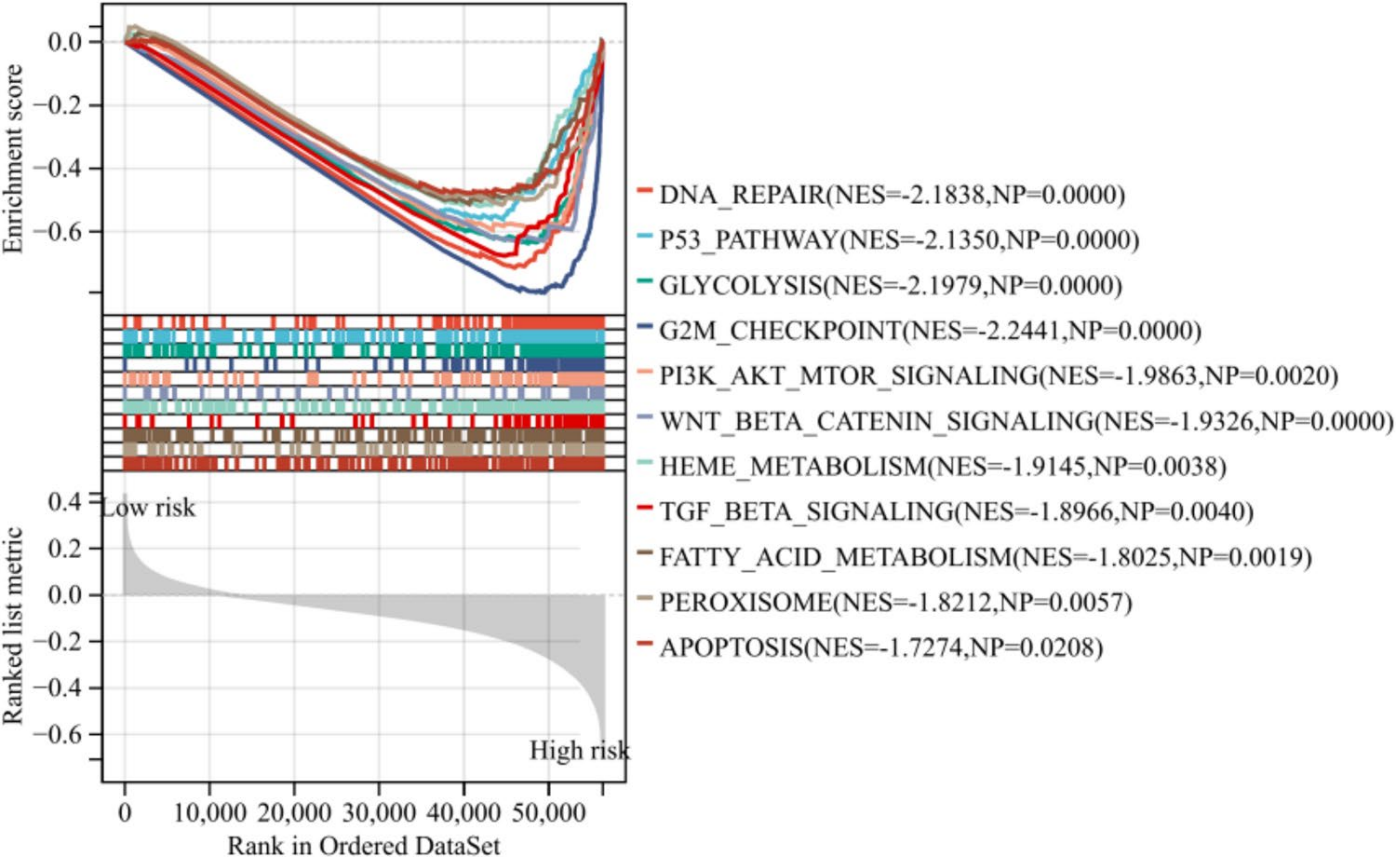
GSEA was conducted to investigate the potential pathways associated with the low-risk and high-risk subgroups

GSEA results revealed that DNA repair, p53 pathway, glycolysis, G2M checkpoint, PI3K-AKT MTOR signaling, TGF beta signaling, fatty acid metabolism, and apoptosis were significantly enriched in the high-risk subgroup (Fig. 6).

*SMURF2* emerged as a significant prognostic indicator for patients diagnosed with oral cancer.

**Fig. 7:**
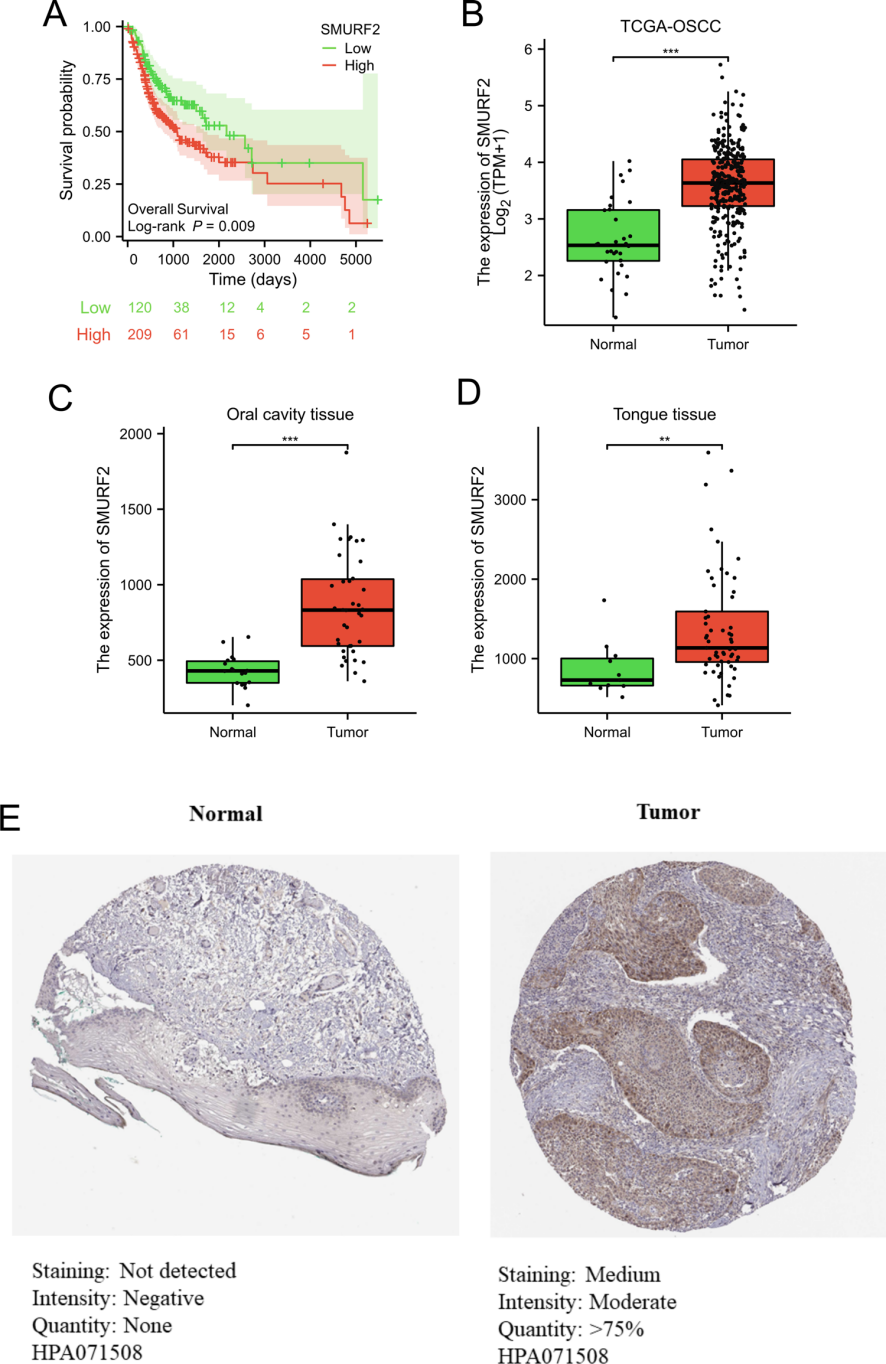
*SMURF2* was a significant predictor of prognosis in patients with oral cancer. **A** Comparison of survival outcomes between OSCC patients classified as high-SMURF2 and low-SMURF2 in the TCGA-OSCC dataset. The expression of SMURF2 was examined in both the TCGA-OSCC dataset (**B**) and through analysis on the TNMplot.com platform (**C**, **D**) to compare the levels between normal and tumor groups. **E** The protein expression level of SMURF2 in both normal and tumor tissue

In OSCC patients, a multivariate regression analysis was conducted on the eight TRGs, revealing that *SMURF2* emerged as an independent prognostic factor (Table 1). The results from Kaplan-Meier analysis indicated a significant correlation between increased *SMURF2* expression and decreased overall survival in patients with oral squamous cell carcinoma (Fig. 7A and Figure S1). The findings from the TCGA-OSCC dataset (Fig. 7B) and analysis conducted on TNMplot.com (Fig. 7C, D) revealed that the mRNA expression level of *SMURF2* was significantly higher in the tumor group in comparison to the normal group (*p* < 0.001 or *p* < 0.01). In addition, by utilizing the HPA database (Fig. 7E), we have also discovered that the protein expression level of SMURF2 exhibited a marked increase in the tumor group as compared to the normal group. As shown in Figure S2, the comparative analysis of SMURF2 gene expression across various cancer types indicated differential expression patterns between normal and tumor tissues. In the case of adrenocortical carcinoma (ACC), the expression of SMURF2 was significantly lower in tumor samples as compared to normal tissue. Similar patterns of reduced SMURF2 expression in tumor samples were observed in kidney chromophobe (KICH), lung adenocarcinoma (LUAD), lung squamous cell carcinoma (LUSC), ovarian serous cystadenocarcinoma (OV), skin cutaneous melanoma (SKCM), testicular germ cell tumors (TGCT), uterine corpus endometrial carcinoma (UCEC), and uterine carcinosarcoma (UCS), all showing statistical significance (*p* < 0.05). Conversely, for cholangiocarcinoma (CHOL), colon adenocarcinoma (COAD), lymphoid neoplasm diffuse large B-cell lymphoma (DLBC), esophageal carcinoma (ESCA), glioblastoma multiforme (GBM), head and neck squamous cell carcinoma (HNSC), kidney renal papillary cell carcinoma (KIRP), acute myeloid leukemia (LAML), brain lower grade glioma (LGG), liver hepatocellular carcinoma (LIHC), pancreatic adenocarcinoma (PAAD), rectum adenocarcinoma (READ), stomach adenocarcinoma (STAD), and thymoma (THYM), tumor tissues demonstrated significantly higher expression levels of SMURF2 when compared to normal tissues (*p* < 0.05).

**Table 1:** Results of multivariate regression analysis

Characteristics	Total (*N*)	HR(95% CI) univariate analysis	*P* value univariate analysis	HR(95% CI) multivariate analysis	*P* value multivariate analysis
ACVR1	329		0.388		
Low	164	Reference			
High	165	1.153 (0.833–1.597)	0.390		
BMP2	329		0.108		
Low	164	Reference			
High	165	0.768 (0.556–1.060)	0.108		
CDK9	329		0.154		
Low	165	Reference			
High	164	1.264 (0.915–1.747)	0.155		
LTBP2	329		0.720		
Low	165	Reference			
High	164	1.061 (0.769–1.463)	0.720		
SLC20A1	329		0.060		
Low	164	Reference		Reference	
High	165	1.361 (0.987–1.878)	0.060	1.165 (0.811–1.675)	0.408
**SMURF2**	329		**0.009**		
Low	165	Reference		Reference	
High	164	1.506 (1.090–2.080)	**0.009**	1.402 (0.975–2.018)	**0.039**
TGFB1	329		0.561		
Low	165	Reference			
High	164	1.100 (0.798–1.516)	0.561		
TGFB3	329		0.246		
Low	165	Reference			
High	164	0.827 (0.600–1.141)	0.247		

Bold indicates statistically significant

### Development of nomogram

**Fig. 8:**
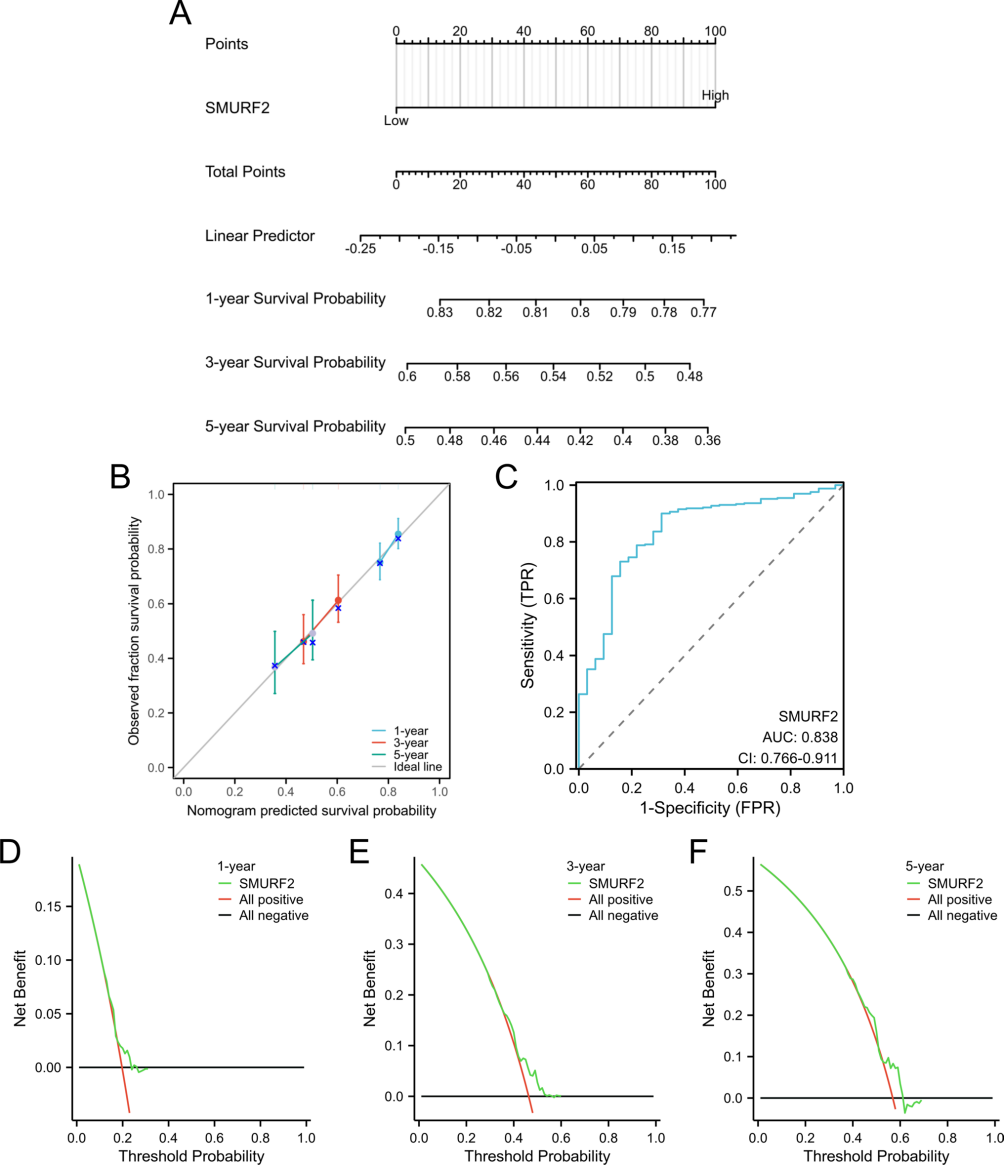
Development and assessment of the nomogram. **A** The nomogram provides the ability to assess the likelihood of survival at 1-year, 3-year, and 5-year time points for individuals with oral cancer. **B** The accuracy of the nomogram in predicting oral cancer patient outcomes is evident in its well-calibrated curve. **C** The diagnostic value of *SMURF2* as a marker for oral cancer is shown through the ROC curve analysis. **D**–**F** Survival rates of oral cancer patients were analyzed using DCA curves for 1, 3, and 5 years, focusing on the SMURF2 gene

We have developed a nomogram to forecast the overall survival rates at 1, 3, and 5 years for patients diagnosed with oral cancer. The nomogram, depicted in Fig. 8A, includes the expression of the *SMURF2* gene. The calibration curve, shown in Fig. 8B, demonstrates a strong correlation between the predicted outcomes and the actual results observed in oral cancer patients. Furthermore, Fig. 8C highlights the high diagnostic accuracy of the *SMURF2* gene, with an area under the curve (AUC) of 0.838. The findings of DCA demonstrated that *SMURF2* exhibited a favorable overall benefit and possessed the ability to forecast the overall survival of individuals diagnosed with oral cancer, both in the short and long term (Fig. 8D–F).

### Single-gene GSEA

**Fig. 9:**
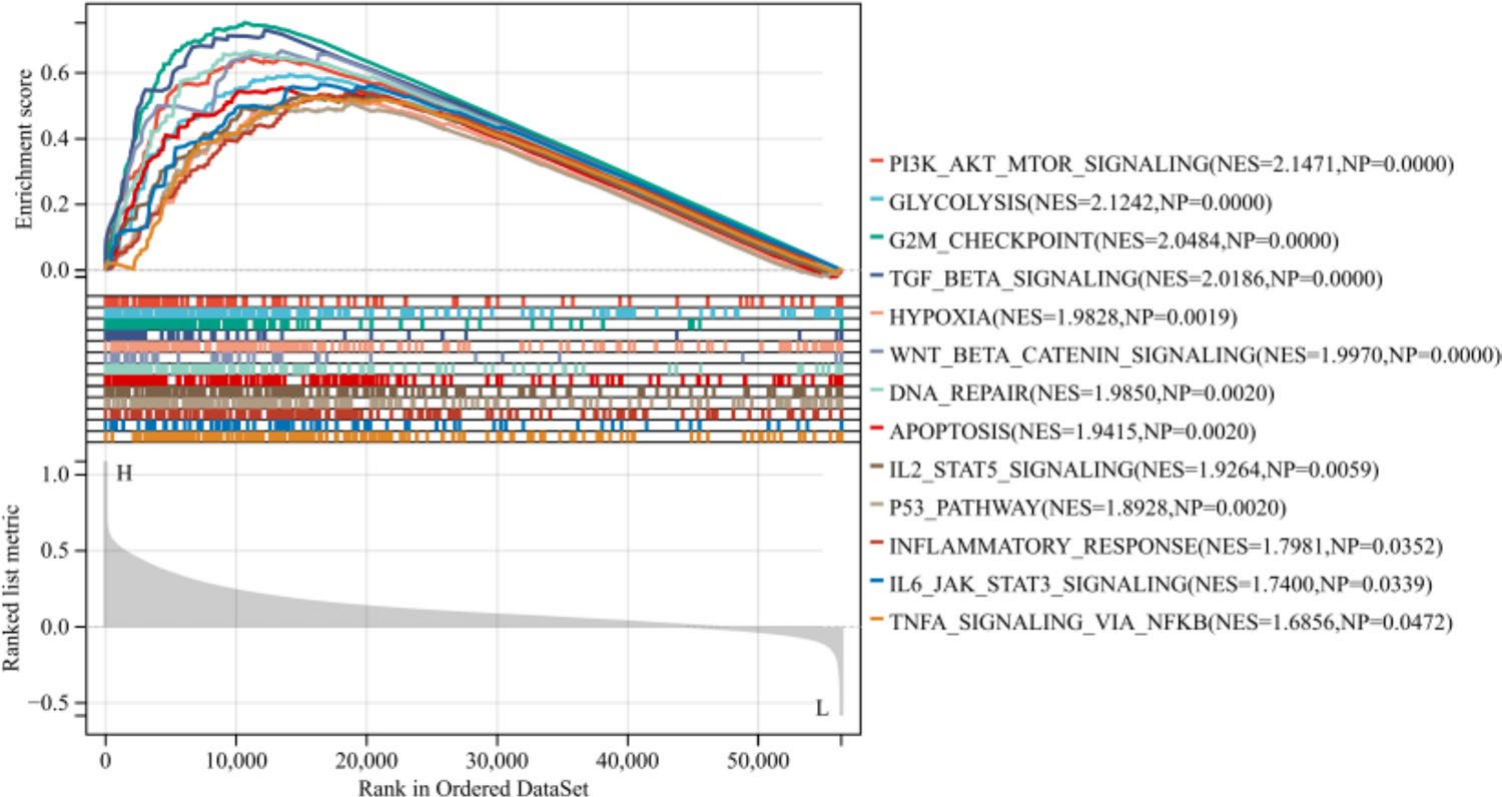
Single-gene GSEA was conducted to investigate the potential pathways associated with the low-SMURF2 and high-SMURF2 subgroups

Single-gene GSEA results revealed that PI3K-AKT MTOR signaling, glycolysis, G2M checkpoint, TGF beta signaling, hypoxia, WNT beta catenin signaling, DNA repair, IL2 STAT5 signaling, p53 pathway, inflammatory response, IL6-JAK-STAT3 signaling, and TNFA signaling via NFKB were significantly enriched in high-SMURF2 subgroup (Fig. 9).

### Investigation of the immune cell infiltration patterns within the subgroups of SMURF2

**Fig. 10:**
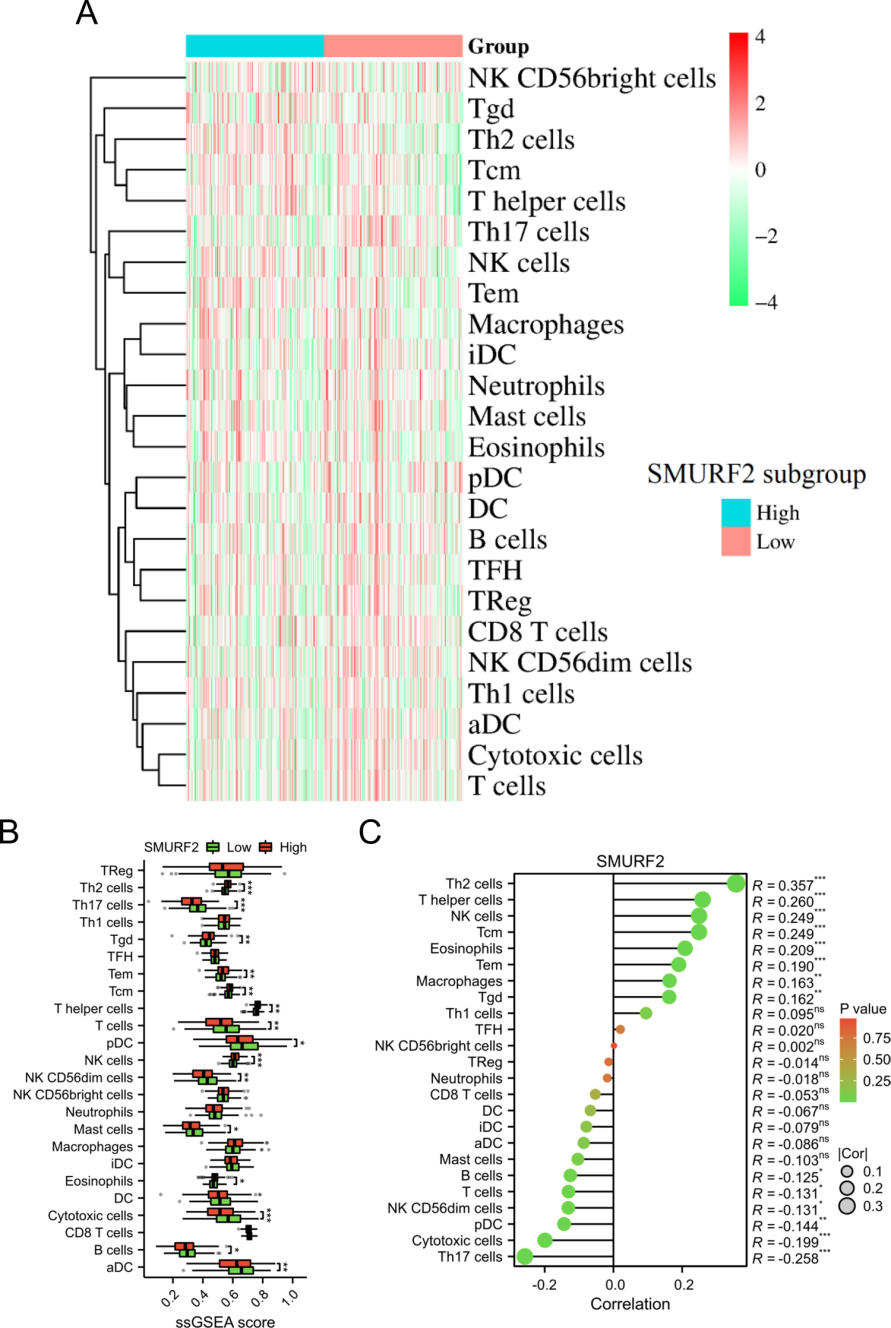
Investigation of the immune cell infiltration patterns within the subgroups of SMURF2. The visual representation of the 24 immune cell types in both the low-SMURF2 and high-SMURF2 groups was presented using a heatmap (**A**) and box plot (**B**). **C** To illustrate the correlation between *SMURF2* expression and immune cell infiltration levels, a Lollipop plot was employed. *p < 0.05; **p < 0.01; ***p < 0.001

As shown in Fig. 10A, B, the ssGSEA results demonstrated significantly different levels of immune cell infiltration between the high and low SMURF2 subgroups. Correlation analysis demonstrated a positive correlation between the *SMURF2* gene and the infiltration level of Th2 cells, T helper cells, NK cells, Tcm, eosinophils, Tem, macrophages, and Tgd. On the other hand, a negative correlation was observed between the *SMURF2* gene and the infiltration level of Th17 cells, cytotoxic cells, pDC, NK CD56dim cells, T cells, and B cells (Fig. 10C).

### Validation of *SMURF2* expression by qRT-PCR analysis

**Fig. 11:**
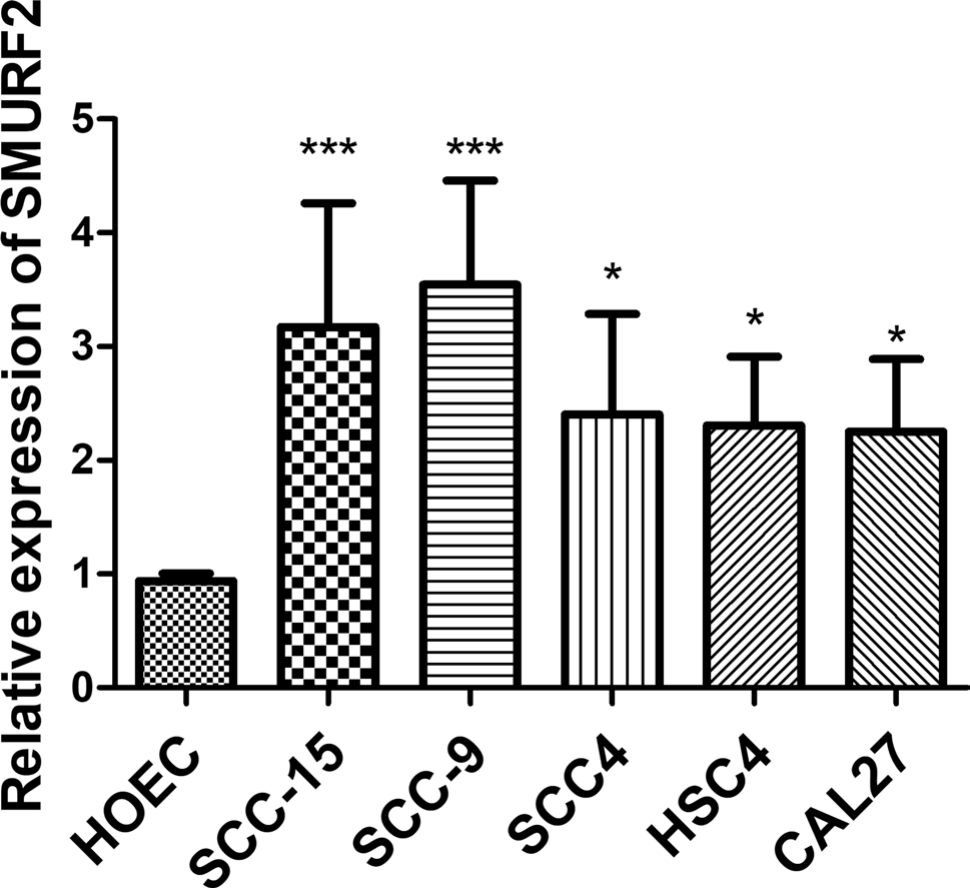
Cell experiments were conducted to validate the expression of *SMURF2*. The expression level of *SMURF2* was compared between various oral cancer cell lines (SCC15, SCC9, SCC4, HSC4, and CAL27) and a human oral gingival epithelial cell line (HOEC cells). *p < 0.05; ***p < 0.001

As shown in Fig. 11, the qRT-PCR findings demonstrated that the expression level of *SMURF2* was increased in SCC15, SCC9, SCC4, HSC4, and CAL27 cells compared to HOEC cells (*p* < 0.05 or *p* < 0.001). These results align with our bioinformatics analyses.

## Discussion

Oral cancer has emerged as one of the most lethal diseases globally. Over the past four decades, there has been little improvement in the outcomes for patients as they continue to suffer from advanced disease, leading to locoregional recurrence and unfavorable results (Gormley et al. 2022). The treatment methods for oral cancer are both expensive and lengthy, making them financially burdensome for many individuals. Therefore, it is crucial to accurately and promptly predict the outcomes of oral cancer, as it can greatly influence the available therapeutic choices and enhance prognosis. The field of diagnosis and prognosis at the individual level holds great promise with the utilization of high-throughput genomics technologies, paving the way for a promising future (Fu et al. 2022; Zhao et al. 2021). The progression of oral cancer involves a complex series of events, in which the TGF-β pathway plays a crucial role (Paterson et al. 2001; Guo et al. 2023). Therefore, the primary objective of this study was to discover a specific signature related to the TGF-β pathway and evaluate the infiltration of immune cells using bioinformatics methods.

The findings from our study indicated that the TGF-β pathway was significantly more active in the oral cancer group compared to the normal group, as demonstrated by the GSVA results. Through an analysis of the TCGA database, we identified 16 specific TRGs that were differentially expressed in patients with OSCC. Furthermore, our study unveiled the close relationship between TGF-β pathway-associated risk subgroups and the prognosis as well as the tumor immune microenvironment of OSCC. These findings are consistent with previous studies. In oral cancer patients, the function of TGF-β signaling is intricate due to its dual effects on tumor development. Initially, TGF-β signaling plays a critical role in suppressing tumor formation. However, in later stages of the tumor, TGF-β signaling can stimulate cancer cell proliferation or facilitate metastatic growth, thus accelerating cancer development (Sun et al. 2008; Qiao et al. 2010). The immune system plays a vital role in the development of cancer. TGF-β and IL-10 are well-known immunosuppressive cytokines that have the ability to dampen immune responses, thereby aiding neoplastic cells in evading detection by the immune system (Arantes et al. 2016; Thomas and Massagué 2005). In OSCC, the immune evasion of NK cells is influenced by the upregulation of TGF-β and IL-10, as well as the downregulation of immune-activating cytokines like NK receptors and IL-2 (Gaur et al. 2014; Dutta et al. 2015). Hence, the impact of the TGF-β pathway on the development of oral cancer could potentially be influenced by its ability to control immune reactions.

Out of the eight TRGs studied, *SMURF2* has emerged as a promising marker with prognostic potential in oral cancer. Examining data from the TCGA database and conducting cell experiments, we discovered that *SMURF2* is significantly upregulated in oral cancer tissues. Importantly, this elevated expression of *SMURF2* was found to correlate with a poorer prognosis in patients with oral cancer. Smad ubiquitin regulatory factor 2 (*SMURF2*), a factor involved in the regulation of ubiquitin protein modification, is primarily found within the nucleus. Its essential function lies in the inhibitory control of TGF-β signaling pathways (David et al. 1835). Increasing evidence suggested that *SMURF2* plays a vital role in regulating tissue homeostasis, establishing cell polarity, maintaining the stability of the genome, and promoting tumorigenesis (Koganti et al. 2018). *SMURF2* plays a crucial functional role in inhibiting cancer cell proliferation and tumorigenesis, achieved by promoting ubiquitination and degradation of several vital cellular proteins (Yu et al. 2020; Li et al. 2019). Elevated *SMURF2* expression levels have been observed in connection with various forms of cancer and have been linked to unfavorable prognosis (Jin et al. 2009; Klupp et al. 2019; Fukuchi et al. 2002). Our study revealed that *SMURF2*, as demonstrated through the analysis of various databases and cell experiments, exhibited increased expression in oral cancer. Furthermore, our findings indicated a direct correlation between elevated *SMURF2* levels and decreased overall survival time in oral cancer patients. These results align with the previously mentioned studies, reinforcing the significant impact of *SMURF2* on prognosis.

Nonetheless, there are limitations to our study. Despite validating the expression level of SMURF2 through cell experiments, the inclusion of a more extensive array of clinical samples would improve the credibility of the findings. Furthermore, the prognostic efficacy of SMURF2 warrants validation in broader cohorts of oral cancer patients.

## Conclusion

To summarize, we have successfully created a prognostic model consisting of eight TRGs for patients with oral cancer. Additionally, this model can also be utilized as a tool to predict prognosis and changes in the immune microenvironment. Furthermore, our research has identified *SMURF2* as a promising prognostic biomarker, showing a significant association with immune cell infiltration in oral cancer. These findings offer a solid foundation for future investigations into the involvement of *SMURF2* in the development and progression of oral cancer.

## Supplementary information

ESM 1

## Data Availability

The website we offer provides access to all the data, and the corresponding author can provide them upon request.
