# Supplementary material for: Transcriptome analysis revealed SMURF2 as a prognostic biomarker for oral cancer
Source: J Appl Genet. 2024 May 3;66(1):155–70. doi: 10.1007/s13353-024-00869-w (PMC11762210; doi:10.1007/s13353-024-00869-w)

Table S1 Sequences of primers used quantitative real-time PCR.

| **Gene** | **Primer** | **Sequence (5'-3')** |
| --- | --- | --- |
| β-actin | Forward | CACGATGGAGGGGCCGGACTCATC |
|  | Reverse | TAAAGACCTCTATGCCAACACAGT |
| SMURF2 | Forward | GTTGTGATGGGTTCTGATTC |
|  | Reverse | CACCAATGGCAAAAGGCT |

Figure S1 Comparison of survival outcomes between OSCC patients classified as high-SMURF2 and low-SMURF2 in the GSE41613 dataset.


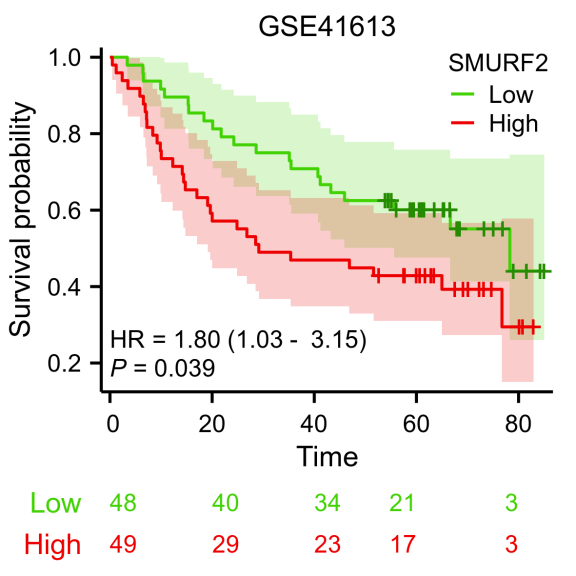


Figure S2 *SMURF2* gene expression levels in human cancers. *p < 0.05, **p < 0.01, ***p < 0.001.


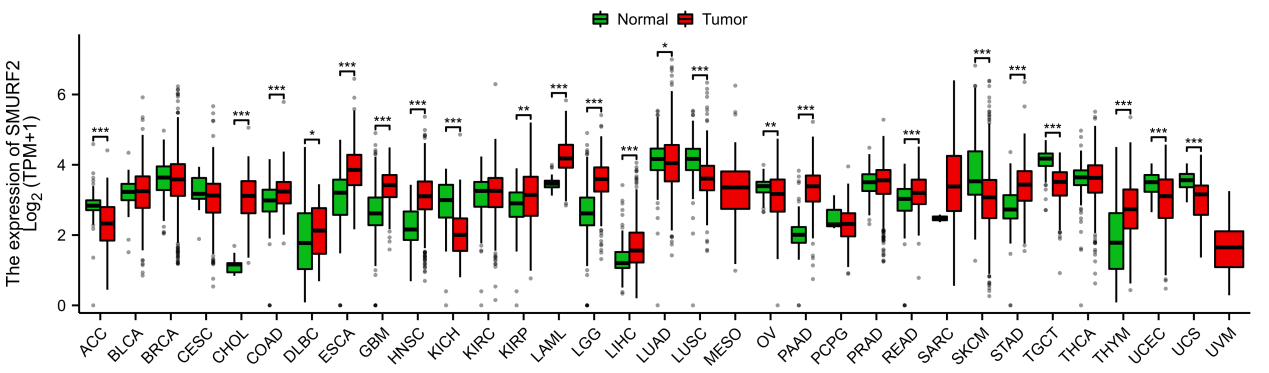

Supplement: Supplementary file 1 — ESM 1 [file 13353_2024_869_MOESM1_ESM.docx]
